# Airway Obstacle Course for Teaching Prehospital Providers Airway Techniques

**DOI:** 10.7759/cureus.18190

**Published:** 2021-09-22

**Authors:** Robert Young, John O Elliott, Brad D Gable

**Affiliations:** 1 Internal Medicine, OhioHealth Doctors Hospital, Columbus, USA; 2 Research, OhioHealth Research Institute, Columbus, USA; 3 Medical Simulation, OhioHealth Riverside Methodist Hospital, Columbus, USA

**Keywords:** skills and simulation training, simulation education, airway mangement, airway intubation, emergency medical service, prehospital first responders, prehospital emergency medicine, obstacle course

## Abstract

Background: Early airway intervention is a vital step in the management of critically ill patients. Emergency medical service (EMS) providers are often first in the chain of survival with equipment to manage airway problems that arise. Therefore, it is paramount that they receive thorough training in aspects of airway management. Often, the training providers currently undergo does not reflect the environmental challenges inherent in EMS. Our obstacle course not only offers trainees a situational environment that simulates common challenges associated with the prehospital environment, but also provides a break from traditional tabletop and lecture-based training methods.

Methods: An airway obstacle course was created that comprised four different “obstacles”. Each obstacle was a patient in a precarious position requiring airway management, and the trainees could manage the obstacles in the order of their choosing. Trainees could choose from four different airway devices based on the local protocol. Once the device was used successfully, it could no longer be implemented in the course, and thus each device was used once. A validated return on the learning model was used for evaluating learning.

Results: Immediately following training, 95.1% (78) trainees felt they were more confident with airway management. Nearly all, 96.4% (79), agreed that the scenarios in the obstacle course were realistic. Participants retained confidence gains in resource management for intubation at the six-month follow-up (p=0.010). In the six months following training, there was a doubling in the number of intubation attempts (24 to 48) and an overall drop in the success rate (75% to 63%). At the six-month follow-up, participants were able to describe specific events where the training helped them with patient management.

Conclusions: The model of an intubation obstacle course as a means of training EMS providers is both realistic to the participants and provides lasting effects to their confidence in resource management skills. Further studies are needed to determine its effects on intubation success rates and patient outcomes.

## Introduction

Prehospital airway management is a cornerstone of emergency medical services (EMS), and success or failure with this process has a significant impact on patient outcomes [1]. The importance of proper training for EMS providers, particularly in skills related to the establishment and maintenance of airway, breathing, and circulation during resuscitation, cannot be overstated. Training for EMS personnel varies widely and may be the major contributor to the widely variable intubation success rates and patient outcomes reported in the literature [2]. Training may involve didactic, task-trainer, or simulation education in addition to performing intubations on actual patients in emergency departments or operating rooms. Typically, these educational opportunities are at table height, in a well-lit and temperature-controlled environment. This is in stark contrast to the dimly lit, resource scarce environment, and often perilously positioned patients that prehospital providers frequently encounter. Creating scenarios and situations that more closely simulate the environments EMS providers often encounter may better prepare them for these difficult situations.

The objectives of this study were to evaluate an airway obstacle course as a novel means for emergency airway management training and to establish a foundation for airway training that focuses on preparing providers for these less than ideal situations.

## Materials and methods

Study design and setting

A validated return on investment in learning model was used to measure training outcomes. This involves five different levels of evaluation: reaction, learning, application and implementation, impact, and return on investment [3]. During the course of this study, we evaluated levels 1 through 3. The [omitted for blinding] Institutional Review Board approved the study. A convenience sample of five fire departments in Central Ohio that share a common medical director were chosen to participate. A total of 82 out of 144 participants had survey responses that were matched and followed from pre-course to post-course surveys, and on to their six-month follow-up survey results. The training took place on location at the respective departments. These departments serve both rural and suburban areas.

Obstacle course

**Figure 1:**
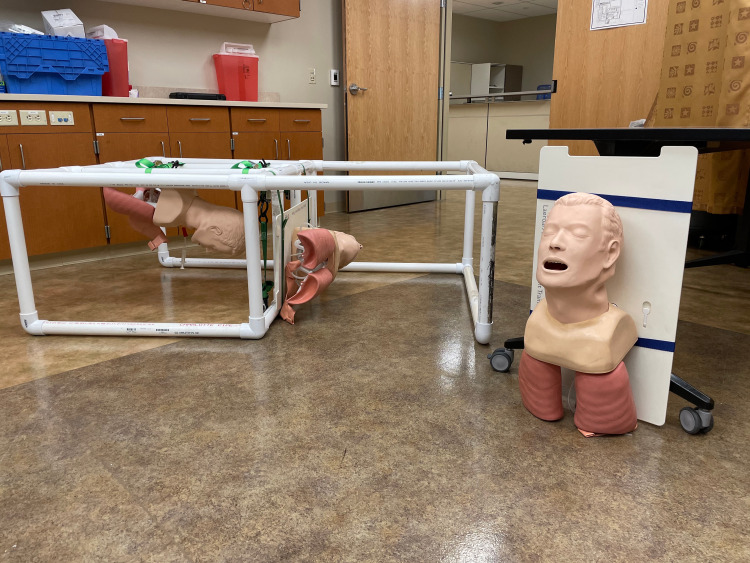
Airway obstacle stations Three obstacle course stations prepared using PVC pipes and airway mannequin heads secured with ratchet straps (not pictured are the fitted covers for blocking out light).

Four obstacle stations made of PVC piping and fitted covers that blocked outside light were constructed using real-world scenarios (Figure 1).

Rooms with low natural lighting were selected at each department to further decrease the light available during training. The first scenario captured the challenges associated with a vehicle rollover accident, where EMS found the patient entrapped upside down in one's car, and had to intubate the victim upside down in an enclosed space. The second station was modeled after a trench collapse where a patient was stuck on his/her side with little space for the provider. The third station was a scenario where a patient was in a motor vehicle accident and was intubated through the windshield by a provider lying on the roof of the car. The fourth and final station replicated a patient that required intubation that was in a confined space and could only be approached from the side.

Participants were given four intubation device options based on those available in their department’s airway protocol. A successful intubation was determined by visualizing bilateral lung inflation with two bag-valve mask breaths. Once they successfully used an airway device at a station, they could no longer use that tool for the course. Participants could choose to perform the four stations in any order. They were given a 12-minute time limit and competed against one another’s times. After participants completed the course, they participated in a debriefing with the department medical director.

Program evaluation strategies

Participants were asked to complete a survey questionnaire prior to the training, immediately following the debriefing, and six months following the training to measure demographics, reactions to the course, and confidence related to airway management skills. Their responses were measured using a five-point modified Likert scale. Statistical analysis was performed using SPSS version 25 (IBM Corp, Armonk, NY).

## Results

Overall, immediately following training, providers reported increases in confidence in every aspect surveyed. Details of their changes in confidence can be seen in Table 1. Specifically, learners reported feeling more confident in devices other than their primary airway management device and in unconventional environments. Participants overwhelmingly agreed that the training was relevant and realistic.

**Table 1:** Participant follow-up survey

Survey details	
Total participants	82
Characteristic	
I feel more confident with airway management in general	
Strongly disagree	0 (0)
Disagree	0 (0)
Neutral	4.9 (4)
Agree	56.1 (46)
Strongly agree	39.0 (32)
I feel more confident with my primary intubation device	
Strongly disagree	0 (0)
Disagree	0 (0)
Neutral	3.7 (3)
Agree	48.8 (40)
Strongly agree	47.6 (39)
I feel more confident with airways devices other than my primary device	
Strongly disagree	0 (0)
Disagree	2.4 (2)
Neutral	11.0 (9)
Agree	62.2 (51)
Strongly agree	24.4 (20)
I feel more confident with airway management in difficult settings (outside of the medic)	
Strongly disagree	1.2 (1)
Disagree	0 (0)
Neutral	8.5 (7)
Agree	50.0 (50)
Strongly agree	40.2 (33)
I feel more confident managing the resources available to me for intubation	
Strongly disagree	0 (0)
Disagree	1.2 (1)
Neutral	1.2 (1)
Agree	62.2 (51)
Strongly agree	35.4 (29)
I feel more confident with time management in critical situations	
Strongly disagree	0 (0)
Disagree	1.2 (1)
Neutral	8.5 (7)
Agree	57.3 (47)
Strongly agree	32.9 (27)
This course was relevant to my work	
Strongly disagree	0 (0)
Disagree	0 (0)
Neutral	0 (0)
Agree	18.3 (15)
Strongly agree	81.7 (67)
This course provided me with new information (or clarified old information)	
Strongly disagree	0 (0)
Disagree	1.2 (1)
Neutral	8.5 (7)
Agree	45.1 (37)
Strongly agree	45.1 (37)
The scenarios presented in the course were realistic	
Strongly disagree	0 (0)
Disagree	2.4 (2)
Neutral	1.2 (1)
Agree	41.5 (34)
Strongly agree	54.9 (45)
I intend to use what I learned from this course	
Strongly disagree	0 (0)
Disagree	0 (0)
Neutral	0 (0)
Agree	30.5 (25)
Strongly agree	69.5 (57)
Overall, I thought the course was	
Very poor	0 (0)
Poor	0 (0)
Neutral	1.2 (1)
Good	14.6 (12)
Very good	84.1 (69)
I thought the instructor(s) were	
Very poor	0 (0)
Poor	0 (0)
Neutral	0 (0)
Good	19.5 (16)
Very good	80.5 (66)

The detailed six-month follow-up survey data can be seen in Table 2. Importantly, it showed lasting improvements in confidence as well as applicability to real-life scenarios. Some participants were able to give specific situations post-training where they were able to apply their knowledge (Table 3).

**Table 2:** Participant six-month follow-up survey results

Characteristic	
How many intubations have you performed since the airway obstacle course? (mean ± SD)	1.3 ± 1.6
I have been able to apply skills from the airway obstacle course to actual patient care	
Strongly disagree	6.1 (5)
Disagree	4.9 (4)
Neutral	37.8 (31)
Agree	36.6 (30)
Strongly agree	14.6 (12)
I feel more confident with my ability to intubate in the field (on scene, not in the ambulance)	
Strongly disagree	1.2 (1)
Disagree	0 (0)
Neutral	15.9 (13)
Agree	62.2 (51)
Strongly agree	20.7 (17)
I feel more confident with airways devices other than my primary device	
Strongly disagree	1.2 (1)
Disagree	1.2 (1)
Neutral	14.6 (12)
Agree	58.5 (48)
Strongly agree	24.4 (20)
I have changed my approach to airway management	
Strongly disagree	2.4 (2)
Disagree	4.9 (4)
Neutral	57.3 (47)
Agree	28.0 (23)
Strongly agree	7.3 (6)
I feel more confident managing the resources available to me for intubation	
Strongly disagree	0 (0)
Disagree	1.2 (1)
Neutral	17.1 (14)
Agree	62.2 (51)
Strongly agree	19.5 (16)
I feel more confident with time management in critical situations	
Strongly disagree	0 (0)
Disagree	2.4 (2)
Neutral	19.5 (16)
Agree	57.3 (47)
Strongly agree	20.7 (17)

**Table 3:** Learner responses at six months when asked to describe the application of knowledge MVC, motor vehicle crash; RSI, rapid sequence intubation; ETT, endotracheal tube

Learner responses to the prompt “Please describe one time when a patient outcome was affected as a result of the training”
Time: Being timed on the multiple scenarios helped gauge how long I'm taking during intubation. We had an MVC and had to RSI. Intubation was a success but kept how long it took in mind.
I'm better at stopping and thinking about the situation instead of a one size fits all approach.
On a run for difficulty breathing we were able to identify the need for airway management early. The options of King Vision, laryngoscope and ETT, and king airway were utilized.
We used all of our airway options that we carry except circ to manage difficult airways recently. This training helped us become more familiar with all our airway options.

Three survey questions were able to be matched immediately post-training and at the six-month follow-up (Table 4). Of the three matched questions, there was a significant lasting improvement in confidence regarding the management of resources available for intubation.

**Table 4:** Comparison of immediate follow-up and six-month follow-up survey responses Analyses were based on the McNemar-Bowker test.

Characteristic	Follow-up	6 months	p-value
I feel more confident with airways devices other than my primary device			0.076
Strongly disagree	0 (0)	1.2 (1)	
Disagree	2.4 (2)	1.2 (1)	
Neutral	11.0 (9)	14.6 (12)	
Agree	62.2 (51)	58.5 (48)	
Strongly agree	24.4 (20)	24.4 (20)	
I feel more confident managing the resources available to me for intubation			0.010
Strongly disagree	0 (0)	0 (0)	
Disagree	1.2 (1)	1.2 (1)	
Neutral	1.2 (1)	17.1 (14)	
Agree	62.2 (51)	62.2 (51)	
Strongly agree	35.4 (29)	19.5 (16)	
I feel more confident with time management in critical situations			0.278
Strongly disagree	0 (0)	0 (0)	
Disagree	1.2 (1)	2.4 (2)	
Neutral	8.5 (7)	19.5 (16)	
Agree	57.3 (47)	57.3 (47)	
Strongly agree	32.9 (27)	20.7 (17)	

## Discussion

The design of the course was meant to simulate low-frequency high-risk situations in airway management with a goal to challenge participants regardless of experience or training. One aspect of the obstacle course trainees found particularly helpful was the ability to manage intubation resources, and this improved confidence was seen not only immediately post-training, but also at the six-month follow-up. This maintenance of confidence in airway management resources is an indicator that an obstacle course offers a unique experience that traditional practice does not. The additional stresses of the training environment such as time limit given, competition between peers, unique obstacles, and darkened environment may help with trainee learning as seen in previous studies [4].

Even though the majority of trainees practice airway management at least yearly and with devices other than their primary, our learners demonstrated significantly improved resource management. The obstacle course encouraged critical thinking and decision making under pressure that may augment skill development. This was reinforced during debriefing where performance was discussed, and application of the gained knowledge may impact airway management in the future. A re-evaluation of skills at six months may help further characterize gains in intubation proficiency.

Multiple EMS providers gave specific examples of how the obstacle course benefitted them while working in the field. Previous research into physician practices and training has shown that a physician’s confidence in his or her procedural abilities is important in multiple aspects of performance and learning [5-7]. After training, participants may have improved their abilities in recognizing when an airway attempt was not working and were more confident in their skills with another method that they previously would not have tried.

Our airway obstacle course was enjoyed by learners and had lasting improvements in confidence at six months. Immediately following the training, learners agreed or strongly agreed that they were more confident in airway management (95%), airway management in difficult settings (83%), airway devices other than their primary device (87%), and resources available for intubation (98%). Learners also felt that the training was realistic (96%) and planned to use what they learned (100%). At six months, learners had applied what they learned while caring for actual patients. Specifically, learners had retained confidence in understanding resources available for intubation (p = 0.01). While learner confidence with airway devices other than their primary trended toward retention at six months, it did not meet statistical significance (p = 0.076). Our airway obstacle course delivers lasting improvements in confidence in airway management and is applied in real-world situations.

Further studies are needed to evaluate the training’s impact on outcomes such as first pass success, rates of complications, and survival to hospital discharge. Comparing this data to national data could shed further light on the utility of an airway obstacle course as a form of airway management training [8].

Limitations

Our study comprised a convenience sample of EMS units that share a medical director. Additionally, our results may not be generalizable to all departments. We did not measure specific outcomes such as time required to intubate or number of attempts. Self-report measures also have inherent social desirability bias, and observational assessments of participant confidence from department officers could strengthen these findings. No patient outcomes were evaluated during this study. Last, we did not control for other confounding variables such as educational interventions and changes in policy/procedure.

## Conclusions

Our airway obstacle course training was well liked by its participants. Following training, EMS providers felt more confident with field intubation, using backup airway devices, resource management, and time management skills. The improvements in resource management were seen immediately after training as well as six months following the course. The knowledge and skills acquired in the training were able to be applied to real-life patient care situations. This study shows that an obstacle course is a novel method of teaching airway management skills and helps prepare EMS providers for low-frequency, high-risk situations.
